# Safety and efficacy of a novel robotic transcranial doppler system in subarachnoid hemorrhage

**DOI:** 10.1038/s41598-021-04751-1

**Published:** 2022-02-10

**Authors:** Kevin Clare, Alan Stein, Nitesh Damodara, Eric Feldstein, Hussein Alshammari, Syed Ali, Christeena Kurian, Jon Rosenberg, Andrew Bauerschmidt, Gurmeen Kaur, Justin Santarelli, Robert Hamilton, Stephan Mayer, Chirag D. Gandhi, Fawaz Al-Mufti

**Affiliations:** 1 New York Medical College School of Medicine, Valhalla, NY USA; 2 Department of Neurosurgery, Westchester Medical Center at New York Medical College, 100 Woods Road, Macy Pavilion 1331, Valhalla, NY 10595 USA; 3 Department of Neurology, Westchester Medical Center at New York Medical College, Valhalla, NY USA; 4 NovaSignal Corp., Los Angeles, CA USA

**Keywords:** Ultrasound, Neurological disorders

## Abstract

Delayed cerebral ischemia (DCI) secondary to vasospasm is a determinate of outcomes following non-traumatic subarachnoid hemorrhage (SAH). SAH patients are monitored using transcranial doppler (TCD) to measure cerebral blood flow velocities (CBFv). However, the accuracy and precision of manually acquired TCD can be operator dependent. The NovaGuide robotic TCD system attempts to standardize acquisition. This investigation evaluated the safety and efficacy of the NovaGuide system in SAH patients in a Neuro ICU. We retrospectively identified 48 NovaGuide scans conducted on SAH patients. Mean and maximum middle cerebral artery (MCA) CBFv were obtained from the NovaGuide and the level of agreement between CBFv and computed tomography angiography (CTA) for vasospasm was determined. Safety of NovaGuide acquisition of CBFv was evaluated based on number of complications with central venous lines (CVL) and external ventricular drains (EVD). There was significant agreement between the NovaGuide and CTA (Cohen’s Kappa = 0.74) when maximum MCA CBFv ≥ 120 cm/s was the threshold for vasospasm. 27/48 scans were carried out with CVLs and EVDs present without negative outcomes. The lack of adverse events associated with EVDs/CVLs and the strong congruence between maximal MCA CBFv and CTA illustrates the diagnostic utility of the NovaGuide.

## Introduction

Each year the United States has 30,000 cases of subarachnoid hemorrhage (SAH), which is consistent with the global incidence rate and represents a significant cause of stroke-related death and disability^1^. Vasospasm is the narrowing of the cerebral arteries and is a common complication of SAH that can lead to delayed cerebral ischemia (DCI), with DCI leading to poor patient outcomes and is associated with morbidity and mortality following SAH^2,3^. Digital subtraction angiography (DSA), and increasingly computer tomography angiography (CTA) are considered the gold standard for determination of cerebral vasospasm as it allows for clear visualization of vasculature^4^. However, repeated imaging from DSA and CTA does not come without risks especially in patients with renal comorbidities. Transcranial doppler (TCD) is a non-invasive bedside modality to rapidly assess vasospasm or stenosis of cerebral arteries following SAH.

TCD offers a safe, relatively inexpensive, and repeatable alternative to CTA to measure cerebral blood flow velocity (CBFv) by insonating proximal cerebral arteries, thus enabling the identification of vasospasm and ischemia^5^. In addition, there are a plethora of potential utilizations of TCD in the neurocritical care setting, such as traumatic brain injury, non-invasive intracranial pressure (ICP) monitoring, large vessel occlusions, sickle cell disease, cerebral malaria, periprocedural monitoring, and even brain stem death^6^. Furthermore, TCD monitoring is also able to predict microemboli burden and as such has shown efficacy for stroke risk stratification after blunt cerebrovascular injury to internal cerebral arteries^7^. Specific thresholds of mean cerebral blood flow have been defined to create criteria for the diagnosis of vasospasm based on TCD readings^8^. However, manual capture of TCD requires a trained sonographer which limits its availability to specific institutions and results in TCD being highly operator dependent. Technicians often have difficulty in finding adequate acoustic windows for ultrasound, leading to vessels not being properly insonated or unsuccessful monitoring attributable to movement of the probe during the procedure^9,10^. Moreover, studies have demonstrated as much as 22.1 cm/s variability between experienced operators^11^.

The NovaGuide (NovaGuide Intelligent Ultrasound, NovaSignal Corp., Los Angeles CA USA) aims to expedite, standardize, and enhance TCD blood flow measurements through combining artificial intelligence and autonomous acquisition that minimizes the need for a trained operator. The NovaGuide system uses a headset that automatically moves the transducer, eliminating variability between users. This allows the system to be used without a trained sonographer while also providing consistent results. We sought to evaluate the safety and validity of the NovaGuide in patients with SAH.

## Methods

This research was conducted at Westchester Medical Center and was approved by New York Medical College Institutional Review Board and the Clinical Research Institute at Westchester Medical Center. All methods were performed in accordance with the relevant guidelines and informed consent was obtained from all subjects and/or their legal guardians. The data will be shared by the principal investigator (F.A.M.) upon reasonable request. We retrospectively evaluated our SAH database to identify patients who underwent NovaGuide imaging between March 1st, 2020 to April 30th, 2020. We collected demographic data including age and sex, admission clinical (modified rankin scale (mRS) and Hunt Hess) and radiologic features. Whether the patient had an external ventricular drain (EVD), central venous line (CVL) or was intubated at the time of their TCD examinations as well as discharge disposition were also collected. Our management protocol of SAH calls for CTA on SAH days 4, 7 and 10–12 (with day 0 being the calendar day of SAH onset). The presence of CTA vasospasm in the M1 segments of the middle cerebral arteries (MCAs) was assess by a neuroradiologist in a standard clinical radiology report, who was blinded to the TCD results. Patients with radiologically confirmed vasospasm (mild, moderate, or severe) were classified as having vasospasm in our analysis.

**Figure 1:**
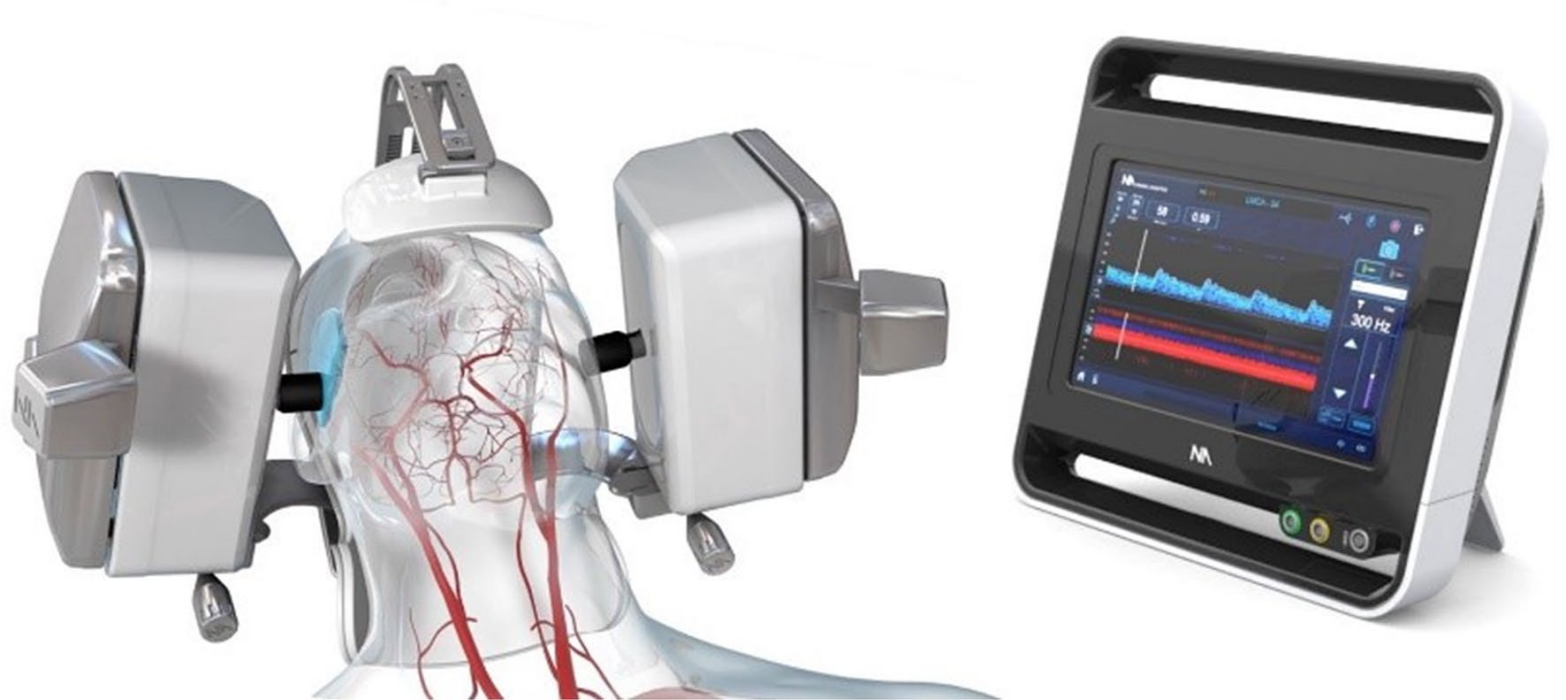
NovaGuide system—five degree of freedom robotic TCD system. [Original image created by manuscript author Dr. Robert Hamilton of NovaSignal].

TCD data was collected using the NovaGuide System purchased from NovaSignal (NovaGuide Intelligent Ultrasound, NovaSignal Corp., Los Angeles CA USA) which uses 2 MHz bilateral probes contained within a five degree of freedom robotic mechanism (Fig. 1). NovaGuide TCD scans were acquired on the same day as the CTAs. All TCD measurements in our unit were acquired with robotic TCD to decrease interoperator variability and expedite acquisition. During the TCD, M-mode and the spectrogram were evaluated to ensure identification and recording of the middle cerebral arteries. Prior to placing the patients head in the head cradle, the two insonators were draped with a plastic cover to protect the patient from the hardware which moves the insonators. Four registration dots were placed with one on the tragus and the other at the lateral canthus, bilaterally. Ultrasound gel was then applied between the area defined by the two registration dots on the ipsilateral side of the head between the tragus and lateral canthus. The patient was put in the supine position and their head was placed in the head cradle. A stabilizer was placed along the frontal bone and attached to the head cradle for additional head stabilization. Once the patient was positioned in the NovaGuide head cradle, the healthcare provider gently engaged the robotic probes onto the patient’s temporal region while receiving real-time feedback from the NovaGuide software regarding the pressure between the probe and the patient’s scalp. Utilizing the cameras on the device, the system captured images regarding the orientation of the patient’s head in the cradle and used machine vision to co-register the patient with the robot using specially designed fiducials. After the registration, a region was identified by the software in which the device searched for the best transtemporal window. The healthcare provider then accepted this search area and the NovaGuide autonomously identified a transtemporal window, located the vasculature of interest (MCA) and performed MCA signal optimization. During this time, NovaGuide used signal quality assessment (SQA) as a rapid means of evaluating signals in real time with segments of data between 350–500 ms^12,13^.

During localization and acquisition of the cerebral blood flow velocity in the MCA, the device samples the velocity across the MCA and selects the area of strongest signal. This ensures the acquired velocities are representative of the current flow dynamics of the MCA. While the MCAs are the only vessel the robotic TCD will autonomously locate and measure, the internal carotid artery terminus anterior cerebral arteries, and posterior cerebral arteries, can be measured manually^14^.

To assess for complications associated with the use of the NovaGuide device, we analyzed electronic medical records for nursing documentation of any EVD or CVL disruptions or removals, skin irritation, or accidental extubations. From the TCD readouts, we collected the maximal and average mean blood flow velocities from the left and right middle cerebral arteries. Average mean velocity ≥ 86 cm/s or a maximum mean flow velocity ≥ 120 cm/s were used as thresholds for defining vasospasm of the middle cerebral artery. After categorizing the velocities, we compared the level of agreement between the NovaGuide TCD measured velocities and CTA reports using a Cohen’s Kappa test. All statistics were run using R-studio and were considered significant when P < 0.05.

## Results

Our study population consisted of twelve patients with two males and ten females with an average age of 63.5 years. From the identified cohort, 50% had radiologically confirmed vasospasm in the cerebral vasculature including the branches of the middle cerebral arteries (66%), the posterior cerebral arteries (17%), and the vertebral arteries (17%) with a mean Hunt Hess score of 2.7. At time of discharge, the average mRS was 3.0 with four patients discharge home, three to in-patient rehabilitation, three to skilled nursing facilities, one to long term acute care facility, and one deceased. Seven of the twelve subjects had an external ventricular drain and central venous line at the time of NovaGuide acquisition. Those seven patients underwent a total of 27 acquisitions using the autonomous robotic device to acquire mean and maximum cerebral blood flow velocities of the middle cerebral arteries. There were no complication associated with damage to the the patient’s skin, EVD, CVL or accidental extubations attributable to the motion of the NovaGuide insonators during velocity acquisition (Table 1).

**Table.1:** Patient characteristics and outcomes. *IP Rehab* In-patient rehabilitation, *SNF* Skilled nursing facility, *LTAC* Long term acute care, *EVD* External ventricular drain, *CVL* Central venous line.

Characteristic	Values:
Median age—yrs (IQR)	63.5 (55–70.25)
Female—no. (%)	10 (84)
Radiologically confirmed vasospasm—no. (%)	6 (50)
Locations	
MCA—no. (%)	4 (66)
PCA—no. (%)	1 (17)
ACA—no. (%)	1(17)
Outcomes:	
Hunt Hess grade	2.7 (1–4)
mRS at discharge	3.0 (1–6)
Discharged to:	
Home	4
IP Rehab	3
SNF	3
LTAC	1
Deceased	1
NovaGuide scans and EVD/CVL complications:	
NovaGuide scans for CTA confirmed MCA vasospasm—no. (%)	17(35.4)
Patients with external ventricular drain & central venous line—no. (%)	7 (58.4)
Total number of NovaGuide scans in EVD & CVL patients—no. (%)	27 (56.2)
EVD/CVL complications—no. (%)	0 (0)
Accidental extubation—no. (%)	0 (0)
Scarification of skin—no. (%)	0 (0)

**Figure 2:**
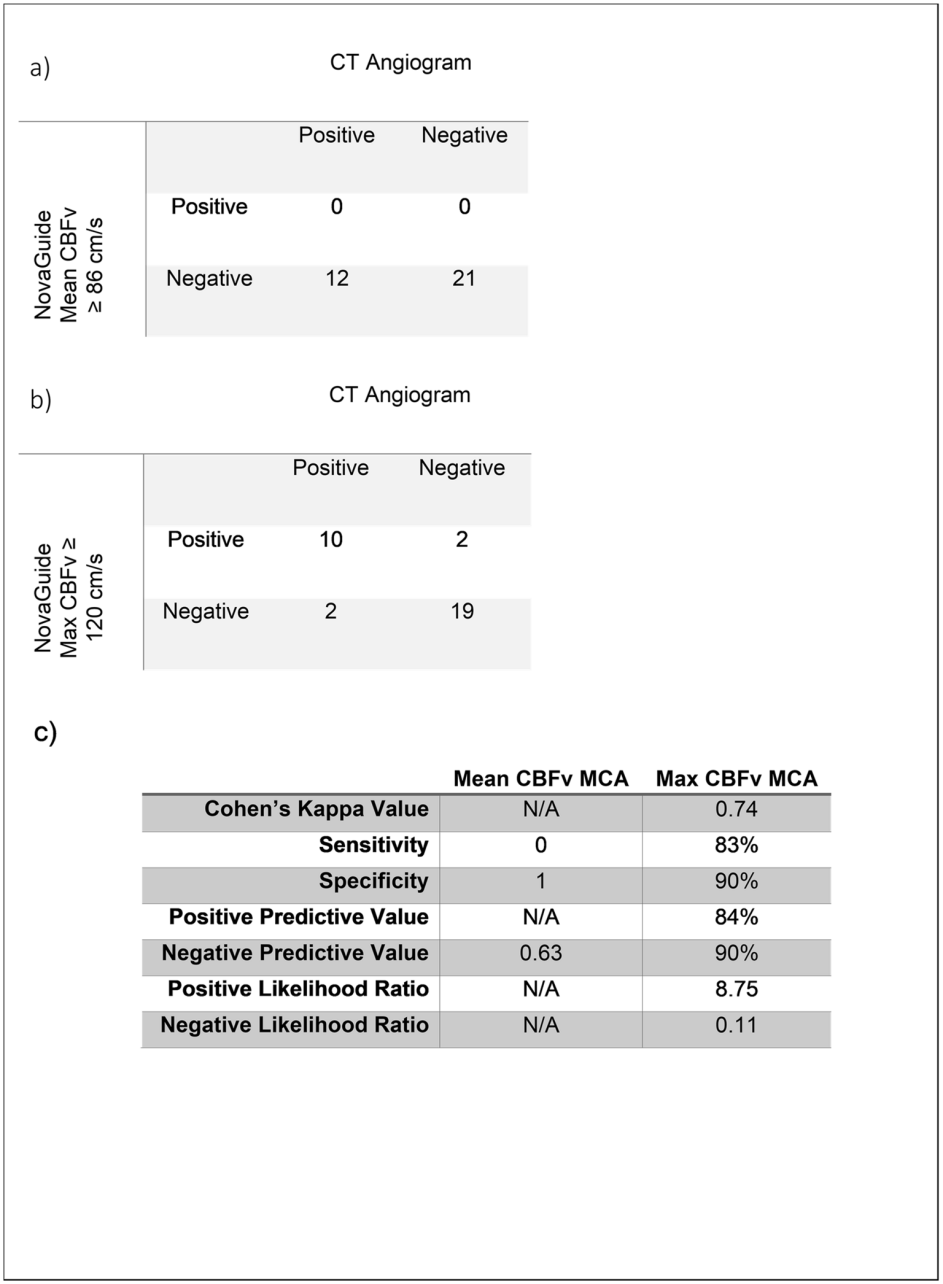
(**a**) Confusion matrices for the outcome of using mean CBFv ≥ 86 cm/s. (**b**) Maximum CBFv ≥ 120 cm/s as a diagnostic metric for determination of vasospasm. (**c**) Parameters quantifying the performance of these metrics.

Using seventeen (m = 17) NovaGuide readings in patients (n = 4) with confirmed vasospasm in the MCAs and sixteen (m = 16) TCD results from patients with no vasospasm (n = 4) the thresholds of the NovaGuide acquired mean CBFv ≥ 86 cm/s or maximum CBFv ≥ 120 cm/s were evaluated for their value as diagnostic criteria. When the mean cerebral blood flow velocity was used as the parameter to predict the presence of MCA vasospasm, no true positive or false positive events occurred (Fig. 2a). Therefore, the Cohen’s Kappa value, sensitivity, positive predictive value (PPV), positive and negative likelihood ratios could not be determined (Fig. 2c). However, when the max mean cerebral blood flow velocity ≥ 120 cm/s was used as the threshold for vasospasm all aspects of the confusion matrix were accounted for (Fig. 2b). Calculation of the Cohen’s Kappa value for the level of agreement between the NovaGuide and the CTA demonstrated substantial inter-observer agreement between the two modalities with a Cohen’s kappa value of 0.74 (P < 0.001). This parameter also had a strong positive and negative predictive value able to indicate the presence of vasospasm in 84% of positive cases and the absence of vasospasm in 90% of negative cases (Fig. 2c). Moreover, using maximum CBFv ≥ 120 cm/s for diagnostic criteria of vasospasm it yielded a positive likelihood ratio (LR +) of 8.75 suggesting that a positive result using this metric means that the patient has ~ 40% increased probability of having vasospasm post positive NovaGuide scan (Fig. 2c).

## Discussion

The present investigation aimed to evaluate the safety and efficacy of the utilization of the NovaGuide system in the setting of a neurocritical care unit to assess for the presents of vasospasm in patients with acute subarachnoid hemorrhage. One of the prominent safety concerns regarding the NovaGuide device is the autonomous guidance of the insonators on the scalp and the risk this motion may pose to external CVL or EVD. We experience no complications associated with the autonomous motion on the EVDs or CVLs in the 27 scans performed. Additionally, we compared the mean and maximum CBFv for the MCA obtained by NovaGuide against CTA confirmed vasospasm. The diagnostic threshold of a maximum CBFv ≥ 120 cm/s produced substantial inter-observer agreement between the NovaGuide and CTA outcomes as well as displayed high sensitivity, specificity, and positive likelihood ratio.

Based on current literature, the consensus among publications is that a normal mean CBFv is below 85 cm/s with any value above this cut-off indicating abnormal flow and vessel stenosis. Specifically for vasospasms, a velocity between 86 cm/s and 120 cm/s is thought to indicate mild vasospasm, velocities from 120 to 200 cm/s as moderate to severe spasm, and greater than 200 cm/s to be critical^15,16^. Thus, we first investigated the mean CBFv as a diagnostic criterion for detecting vasospasm using the NovaGuide acquired flow velocities. Surprisingly, there were no true positives detected when using this criterion.

It is highly unlikely that this outcome is due to the NovaGuide system as it is an effective tool for acquiring MCA CBFv. Some studies have documented that even highly trained sonographers can have difficulty in correctly identifying the MCA, misidentifying it as often as 40% of the time^17^. However, in the study by O’Brien et al. the NovaGuide matched the efficacy in MCA signal acquisition of a registered vascular technician (RVT) on 86 healthy subjects with a mean velocity accuracy of 99.7% (95% CI 97.7%–101.7%) with respect to the RVT.This investigation also showed a mean time to signal acquisition of 74.4 s (95% CI 60–90 s) and a no-window rate of 3.5% (RVT no-window rate was 4.1%)^18^. Additional investigations utilizing the NovaGuide system have reported similar rates of accuracy in identifying and measuring the MCA with the study by Esmaeeli et al. reporting a concordance correlation coefficient of 0.83 between manual acquired TCD velocities and the NovaGuide. Taken together, these studies demonsrate the NovaGuide’s ability to reliability and accurately identify and acquire MCA velocities^19^.

Our outcome of no true positives when using mean CBFv to determine presence of vasospasm might be attributed to the age of our patient population as previous studies have documented the decline of mean CBFv with age^19–21^. Thus, the lack of results using the mean CBFv as diagnostic criteria may reflect that in our patient population the baseline mean CBFv was low. Therefore, when vasospasm did occur as documented by angiography, the rise in CBFv was not great enough to reach or surpass threshold.

Interestingly, when we applied the mean CBFv threshold for mild vasospasm to the maximum CBFv (Max CBFv ≥ 120 cm/s) from the NovaGuide data we obtained a performance comparable to previous publications. A meta-analysis of 26 studies comparing TCD against angiography found that utilizing the criteria of mean CBFv > 120 cm/s TCD had a sensitivity of 67% (48–87%), specificity of 99% (99–100%), positive predictive value of 97% (95–98%), negative predictive value of 78% (65–91%), positive likelihood ratio of 17 (5–56) ,and negative likelihood ratio of 0.4 (0.2–0.7)^14^. Our metrics of sensitivity (83%), negative predictive value (90%), and positive likelihood ratio (8.75) are contained within these reported ranges. Additionally, other groups have reported specificities consistent with our results^6,22^. Taken together, this suggests that maximum CBFv ≥ 120 cm/s may have some diagnostic value in determining the presence of MCA vasospasm.

## Conclusion

Although this study yields valuable insight into the usage of the NovaGuide system for determination of cerebral vasospasm in acute SAH, this study is not without limitations. Mainly, the sample size of the current investigation is small with safety being evaluated in seven patients and the detection of MCA vasospasm in four. The low number of patients for safety however is partially offset by the total of 27 individual scans performed. Thus, additional work is necessary to substantiate our claim of the safety of the NovaGuide device as well as the efficacy of using maximum CBFv ≥ 120 cm/s as a diagnostic threshold for MCA vasospasm. Future studies will focus on enrolling additional patients, evaluating the relationship between ICP and the Pulsatility Index (PI), and safety and efficacy of this device on patients managed with craniotomy and clipping for control of aneurysmal SAH.

## Data Availability

The data that support the findings of this study are available from the corresponding author upon reasonable request.
